# A Cross-Region Meta-Analysis and Machine Learning Identifies a 37-Gene Signature Associated with Alzheimer’s Disease

**DOI:** 10.3390/biomedicines14092032

**Published:** 2026-09-10

**Authors:** Kashvi Chirag Shah, Ethan Littlestone, Md Roungu Ahmmad, Chunmei Wang, Yong Xu, Xiaoli Zhang

**Affiliations:** 1 USF Health, College of Nursing, University of South Florida, Tampa, FL 33612, USA; kashvichirag@usf.edu (K.C.S.); elittlestone@usf.edu (E.L.); ahmmad@usf.edu (M.R.A.); 2 USDA/ARS Children’s Nutrition Research Center, Department of Pediatrics, Baylor College of Medicine, Houston, TX 77030, USA; chunmei.wang@bcm.edu; 3 USF Health, College of Medicine, University of South Florida, Tampa, FL 33612, USA; yongxu@usf.edu

**Keywords:** Alzheimer’s disease, transcriptomics, brain regions, meta-analysis, differential gene expression, Braak staging, biomarker discovery, machine learning

## Abstract

**Background/Objectives**: Alzheimer’s disease (AD) shows marked transcriptomic heterogeneity across brain regions, limiting reproducibility. We aimed to identify robust cross-region gene signatures using meta-analysis. **Methods**: Differential expression (limma-voom) was performed on five bulk RNA-seq datasets (n = 230; 154 AD donors, 76 controls) from the hippocampus to cortical regions. Consensus DEGs were identified via Stouffer’s Z, random effects, and MetaVolcanoR models. Pathway enrichment and associations with Braak stage were evaluated. Validation was conducted in two independent cohorts, with predictive performance assessed using machine learning. **Results**: Thirty-seven consensus DEGs (16 up, 21 down; FDR ≤ 0.05) were identified across ≥4 datasets. The enrichment results revealed increased expression of glial and ECM-associated genes and decreased expression of synaptic and GABAergic genes. Thirty-six of 37 genes correlated with Braak stage, with all 37 remaining significantly associated after covariate adjustment. The signature predicted AD with AUCs of 0.784 and 0.861 for validation in two independent cohorts. **Conclusions:**: We identified a consistent cross-region signature linking synaptic and glial changes to neuropathological severity, highlighting new mechanisms and potential biomarkers.

## 1. Introduction

Alzheimer’s disease (AD) is the most prevalent neurodegenerative disorder and the leading cause of dementia, affecting over 55 million people worldwide and 7.2 million Americans aged 65 and older [1,2]. It represents a major and escalating public health crisis, with cases projected to nearly triple by 2060 as the global population ages [3]. AD is characterized by progressive cognitive decline, memory impairment, and behavioral disturbances. It is accompanied by neuropathological hallmarks, including the extracellular accumulation of amyloid-β (Aβ) plaques, the formation of intracellular neurofibrillary tangles composed of hyperphosphorylated tau, neuroinflammation, and widespread synaptic and neuronal loss [4]. However, despite decades of intensive research, the molecular mechanisms underlying AD pathogenesis remain incompletely understood, and effective disease-modifying therapies remain elusive [5].

The progression of AD pathology follows a stereotypical spatial and temporal pattern, defined by the Braak staging system, which maps tau pathology and disease severity from the transentorhinal region (I–II) to the limbic (III–IV) and neocortical (V–VI) areas [6,7]. Importantly, recent studies have demonstrated that transcriptomic changes such as early increases in synaptic activity can precede plaque pathology [8]. Therefore, linking gene expression changes to Braak stages is critical for identifying stage-associated molecular features and therapeutic targets.

A major challenge in elucidating AD pathophysiology lies in its molecular heterogeneity across individuals and brain regions. Transcriptomic profiling of postmortem brain tissue has emerged as a powerful approach to characterize the molecular landscape of AD [9]. Numerous independent studies have profiled gene expression in AD-affected brain regions, revealing alterations in synaptic function, neuroinflammation, lipid metabolism, and cellular stress responses [10,11,12], as well as sex-specific patterns with altered transcriptomics in different brain regions of different genders [13,14,15]. However, individual studies are often limited by modest sample sizes, regional specificity, technical variability [16], and biological heterogeneity, which can result in inconsistent findings and reduced statistical power to detect true disease-associated signals [17].

Meta-analysis represents a systematic approach to integrate findings across multiple independent studies from diverse cohorts and brain regions to identify robust and reproducible molecular signatures and consistent disease-associated transcriptional changes [18]. Several complementary meta-analytic strategies exist, including *p* value combination methods, effect size pooling through random-effects models, and rank-based aggregation approaches, each with distinct statistical properties and assumptions [17,19]. Previous meta-analyses of AD human brain transcriptomics have revealed consistent dysregulations: downregulated synaptic transmission and nervous system development genes and upregulated immune response genes [17,18,20]. However, these studies have generally relied on a single meta-analytic method, often focusing on individual brain regions or a limited subset of regions, restricting the generalizability and reproducibility of their findings [10,13,18,21]. Moreover, few studies have systematically characterized stage-dependent molecular changes related to neuropathological progression [10,22,23].

To address these limitations, we performed a multi-method meta-analysis across five brain regions and datasets. We identified reproducible molecular signatures and dysregulated pathways linked to AD pathology and Braak stages using this method and further validated their predictive accuracy in independent datasets. This integrative approach provides a refined transcriptional portrait of AD, highlighting high-confidence candidate genes for disease mechanisms and therapeutic development.

## 2. Materials and Methods

### 2.1. Datasets

We utilized five publicly available human brain RNA-seq datasets from the Gene Expression Omnibus (GEO): GSE203206, GSE67333, GSE95587, GSE159699, and GSE278723 for meta-analysis. For each dataset, only samples annotated as clinically diagnosed AD or age-matched cognitively normal controls were retained for analysis. Samples labeled as mild cognitive impairment (MCI), other neurodegenerative conditions, or missing diagnostic metadata were excluded. These datasets represent diverse brain regions implicated in AD pathology, including the lateral temporal cortex (GSE159699), hippocampus (GSE67333), occipital lobe (GSE203206), and fusiform gyrus (GSE95587). The GSE278723 dataset comprised transcriptomic profiles from hippocampal subregions (CA1-CA4) and the entorhinal cortex. While demographic and diagnostic annotations (age, sex, and region) were available across all datasets, Braak staging information was only provided for a subset of studies. Sequencing batch was not annotated in the deposited metadata for any dataset. GSE125583 (fusiform gyrus) and Mount Sinai Brain Bank (MSBB) study (syn27068756) encompassing superior temporal gyrus, frontal pole, parahippocampal gyrus, prefrontal cortex, and inferior frontal gyrus were used for validation.

### 2.2. Data Processing and Differential Expression Analysis

RNA-seq counts data were assembled into *DGEList* objects using the *edgeR* package (v4.10.1) and normalized using the Trimmed Mean of M-values (TMM) method. Filtering was applied to filter out noise level-expressed genes if log_2_ counts per million (CPM + 1) ≥ 1 in at least as many samples as the smaller comparison group. For example, with 8 controls and 39 AD samples, the minimum threshold was set to 8, meaning that a gene was only retained if it had an expression of log_2_ (CPM + 1) ≥ 1 in at least 8 samples across the dataset.

To ensure consistency across the datasets, gene identifiers were standardized to HGNC gene symbols. Ensembl IDs were mapped to gene symbols using the *org.Hs.eg.db* annotation package (v3.23.1). For each dataset, differential expression (DE) analysis between the AD and control samples was performed using the limma-voom workflow (*limma *v3.66.0). For each dataset, differential expression was assessed using a linear model with diagnosis (AD versus control) as the sole predictor (design: ~diagnosis). A consistent minimal model was used across all five datasets to maintain comparability of effect estimates for meta-analysis; covariate-adjusted sensitivity models are described in Section 2.3.

Genes with unadjusted *p* ≤ 0.05 and |log_2_FC| > 0.58 within each dataset were carried forward as candidate inputs to the meta-analysis. This threshold served as a permissive screening criterion rather than a test of statistical significance; formal inference and multiple-testing correction were performed at the meta-analytic stage. Age and sex were subsequently included as covariates in the Braak-stage association analyses, in which samples were pooled and analyzed jointly.

For GSE278723, each donor contributed five samples spanning hippocampal subregions CA1–CA4 and the entorhinal cortex. Because these subregions differ in their vulnerability to AD pathology, differential expression was analysed independently within each subregion using the workflow described above, preserving region-specific effects. For each gene, the five region-level log_2_ fold changes were then combined into a single study-level estimate by inverse-variance weighting, with each region weighted by the reciprocal of its squared standard error; the pooled estimate and its standard error. Between-subregion heterogeneity was quantified for each gene using Cochran’s Q and the I^2^ statistic across the five regions prior to pooling. This two-stage design ensures that GSE278723 enters the cross-study meta-analysis as one study-level effect estimate, on equal footing with the other four datasets.

For the MSBB study, samples with a Braak score ≥ 4 and a CERAD 2 or 3 are labelled as AD whereas those with a Braak score < 4 and a CERAD score of 1 or 4 were labeled controls. Both the MSBB and GSE125583 datasets were z-score normalized prior to validation.

### 2.3. Meta-Analysis to Identify Consensus Differentially Expressed Genes

To identify consensus differentially expressed genes across different studies, we applied three complementary meta-analytic approaches: a direction-aware Stouffer Z-method [19] that computed signed Z-scores from per-study *p* values and logFC direction, weighted by the square root of sample size; DerSimonian–Laird random-effects models [24] fitted using R package metafor (v4.8.0) (yi = logFC; vi = SE^2^) for genes with available standard errors; and Fisher’s combined probability test and mean fold change summarization method using the combining_mv function in MetaVolcanoR (v1.0.1) [25].

From the above meta-analyses, genes were classified as “consensus” when they satisfied all the following criteria:

(i) Detected in four or more datasets;
(ii) Exhibited consistent direction of differential expression across studies;
(iii) Were significant in all three meta-analytic frameworks (direction-aware Stouffer FDR ≤ 0.05, random-effects FDR ≤ 0.05, and MetaVolcano FDR ≤ 0.05);
(iv) Exhibited low inter-study heterogeneity (I^2^ < 40%);
(v) Remained significant in leave-one-study-out sensitivity analysis (raw, unadjusted maximum leave one-study-out *p* < 0.05), in which each dataset was sequentially omitted and the remaining studies were re-pooled using the same random-effects model; GSE278723, comprising all five hippocampal–entorhinal subregions, was omitted as a single study.

Three sets of sensitivity analyses were performed. The first sensitivity analysis assessed the effect of the per-study screening criterion by repeating the meta-analysis using two alternative gene-selection approaches. In the primary analysis, genes were eligible if they met an unadjusted *p* ≤ 0.05 and |log_2_FC| > 0.58 within each dataset. In the first specification, no per-study *p*-value or fold-change threshold was applied; all expression-filtered genes quantified in at least four datasets were retained. In the second specification, the per-study unadjusted *p*-value threshold was replaced with a Benjamini–Hochberg FDR ≤ 0.05, while the |log_2_FC| > 0.58 threshold was retained. For each specification, eligible genes were subjected to the same downstream analysis pipeline as the primary analysis, including region-level collapsing, application of the three meta-analytic models, and leave-one-dataset-out sensitivity analysis.

In the third and fourth specifications, the per-study significance threshold was retained at the primary setting (unadjusted *p* ≤ 0.05), while the fold-change threshold was varied to |log_2_FC| > 1.00 (2-fold) and |log_2_FC| > 0.25 (approximately 1.19-fold), respectively. These analyses therefore assessed sensitivity to the fold-change criterion, whereas the first two specifications assessed sensitivity to the significance threshold. All four specifications were applied to the unfiltered per-dataset differential expression results and processed through the same downstream pipeline as the primary analysis, including the three meta-analytic frameworks and leave-one-dataset-out sensitivity analysis. As a pipeline check, the analysis was also repeated using the primary |log_2_FC| > 0.58 threshold and reproduced the primary result.

The second sensitivity analysis assessed potential residual confounding by available demographic and technical covariates. Differential expression analyses were refitted within each discovery dataset using the covariates reported for that dataset. Models included diagnosis, age, and sex, with post-mortem interval (PMI), RNA integrity number (RIN), and *APOE* genotype additionally included when available for at least 90% of samples. Covariate adjustment therefore varied across datasets according to data availability; sequencing batch information was not available for any dataset. Within each dataset, adjusted and unadjusted models were fitted to the same set of samples, and the 37 consensus genes were compared with respect to effect direction and statistical significance. For GSE278723, subregion-specific estimates were pooled to generate a single study-level estimate, as described in Section 2.2. Concordance and significance were assessed using four thresholds: nominal *p* < 0.05; nominal *p* < 0.05 with |log_2_FC| > 0.58; FDR < 0.05; and FDR < 0.05 with |log_2_FC| > 0.58.

### 2.4. Downstream Harmonization and Neuropathological Association Analyses

To test the association between gene expression and Braak stages, the four datasets containing BRAAK staging information were integrated, ComBat batch correction (*sva *v3.56.0) was applied using log_2_ (CPM + 1) values while preserving biological covariates (PATHDX, AGE, SEX), and the expression values were further z-score normalized. Associations between adjusted gene expression and Braak stage were examined via three complementary approaches: (i) Spearman correlation with numeric Braak stages (0–6), (ii) the Braak stage was dichotomized into Low (≤3) and High (>3) groups, and then one-way ANCOVA was performed for each gene on z-score normalized expression to test whether there was a significant difference in the gene expression between the high and low Braak stages, adjusting for brain region, age, and sex. (iii) Logistic regression was further used to assess the associations between increased gene expression and high Braak stage, adjusting for brain region, age, and sex. Odds ratios (OR) with 95% confidence intervals were calculated by exponentiating the regression coefficients and their respective boundary values of the gene. Within each region, we also tested whether gene expression was associated with Braak stages using ANCOVA, adjusting for age and sex.

### 2.5. Pathway Enrichment Analysis

Functional enrichment analyses of the DEGs from each individual dataset as well as the consensus gene list from the meta-analysis were performed using the *clusterProfiler *library (v4.20.0) [26], separated by up- and downregulated genes, to identify perturbed biological processes within each brain region as well as across brain regions in AD donors [26]. Pathway enrichment was performed using Gene Ontology (GO) categories, including Biological Process (BP), Molecular Function (MF), and Cellular Component (CC) and Kyoto Encyclopedia of Genes and Genomes (KEGG) pathway enrichment. Over-representation analysis was performed using the default *clusterProfiler* background of all annotated human genes, with Benjamini–Hochberg correction for multiple testing.

Protein–protein interaction (PPI) analysis of the consensus genes was performed using the STRING database (v12.0). All analyses were conducted in R (v4.6.0). Statistical significance was defined as FDR-adjusted *p* ≤ 0.05 unless otherwise specified.

### 2.6. Validation of the Consensus Genes in Independent Cohorts

The validation performance of the identified DEGs was evaluated in two independent cohorts: MSBB and GSE125583. Because MSBB donors contributed samples from multiple Brodmann areas, cross-validation was performed at the donor level using StratifiedGroupKFold, ensuring that all samples from the same donor were assigned to a single fold. In GSE125583, folds were defined at the sample level because each donor contributed only one sample. The gene signature was defined exclusively from the five discovery datasets and was fixed prior to any analysis of the validation cohorts; no feature selection, ranking, or filtering was performed within either validation cohort. An elastic-net logistic regression model (L1 ratio = 0.5, C = 1.0) with balanced class weighting was trained and evaluated using donor-grouped stratified 5-fold cross-validation as the primary evaluation strategy; leave-one-out cross validation (LOOCV) was additionally performed as a supporting sensitivity analysis. Both were implemented using scikit-learn (v1.9.0) in Python (v 3.14.4) [27]. Hyperparameters were specified a priori and were not tuned using validation data; no grid search or model selection was performed in either cohort.

To prevent information leakage, all preprocessing and model fitting were encapsulated within a scikit-learn *Pipeline* executed inside each cross-validation partition. Gene-wise standardization parameters (mean and standard deviation) were estimated from the training partition only and applied to the held-out samples, and model coefficients were likewise estimated solely from training partitions. Held-out predicted probabilities were pooled across folds to compute a single ROC curve and AUC per cohort. Confidence intervals were calculated by bootstrap resampling at donor level in MSBB and sample level in GSE125583. Because model coefficients were estimated separately within each cohort, the prespecified gene signature was evaluated using within-cohort cross-validation rather than external model validation.

## 3. Results

### 3.1. Summary of the Datasets Used for Analysis

Five independent RNA-seq studies encompassing a total of 230 brain samples (154 AD donors and 76 controls) were included in the meta-analysis. The study characteristics of each dataset are summarized in Table 1, while demographic characteristics by diagnostic group and the availability of clinical and technical metadata are provided in Table S1. The datasets spanned five brain regions, namely, the hippocampus, lateral temporal cortex, occipital lobe, fusiform gyrus, and entorhinal cortex, which represent distinct stages of Alzheimer’s disease neuropathology.

There were 94 female and 136 male samples whose ages ranged from 41–103 years. Another two datasets, MSBB and GSE125583, were used for validation. The MSBB data included 607 AD samples and 436 controls covering regions of the superior temporal gyrus, frontal pole, parahippocampal gyrus, prefrontal cortex, and inferior frontal gyrus, whereas GSE125583 included 158 AD donors and 42 controls from the fusiform gyrus.

### 3.2. Differential Gene Expression and Pathway Analysis for Individual Datasets

The differentially expressed genes (DEGs) were identified from each individual dataset by comparing the expression of genes in AD samples to the corresponding controls in each dataset. The number of DEGs (unadjusted *p* ≤ 0.05, |log_2_FC| > 0.58) identified from each dataset varied among the datasets, ranging from 261 to 2291, as shown in Table 1 and Table S2a–e. The top DEGs from each dataset are displayed in volcano plots and heatmaps (Figure S1A–E). Region-level effect estimates within GSE278723 were highly concordant across the hippocampal subfields and entorhinal cortex (median I^2^ = 0%; 99.2% of genes has I^2^ < 40%, with no significant heterogeneity after FDR correction Table S3).

We next performed pathway enrichment analysis for the DEGs from each dataset. Pathway analyses from independent datasets showed convergent dysregulation of synaptic and glial processes across brain regions (Table S4). KEGG analysis consistently identified downregulation of neurotransmission-related pathways, including axon guidance, neuroactive ligand–receptor interaction, GABAergic synapse, synaptic vesicle cycle, calcium signaling, and broader neurodegeneration pathways, in at least two datasets, whereas GO terms recurrently highlighted upregulation of glial and extracellular matrix (ECM)-related processes (e.g., gliogenesis, glial cell development, collagen-containing ECM, focal adhesion, lysosomal lumen) and downregulation of synaptic vesicle, postsynaptic membrane, vesicle transport, ion channel, and neurotransmitter receptor functions.

### 3.3. Cross-Study Meta-Analysis and Consensus Gene Identification

**Figure 1:**
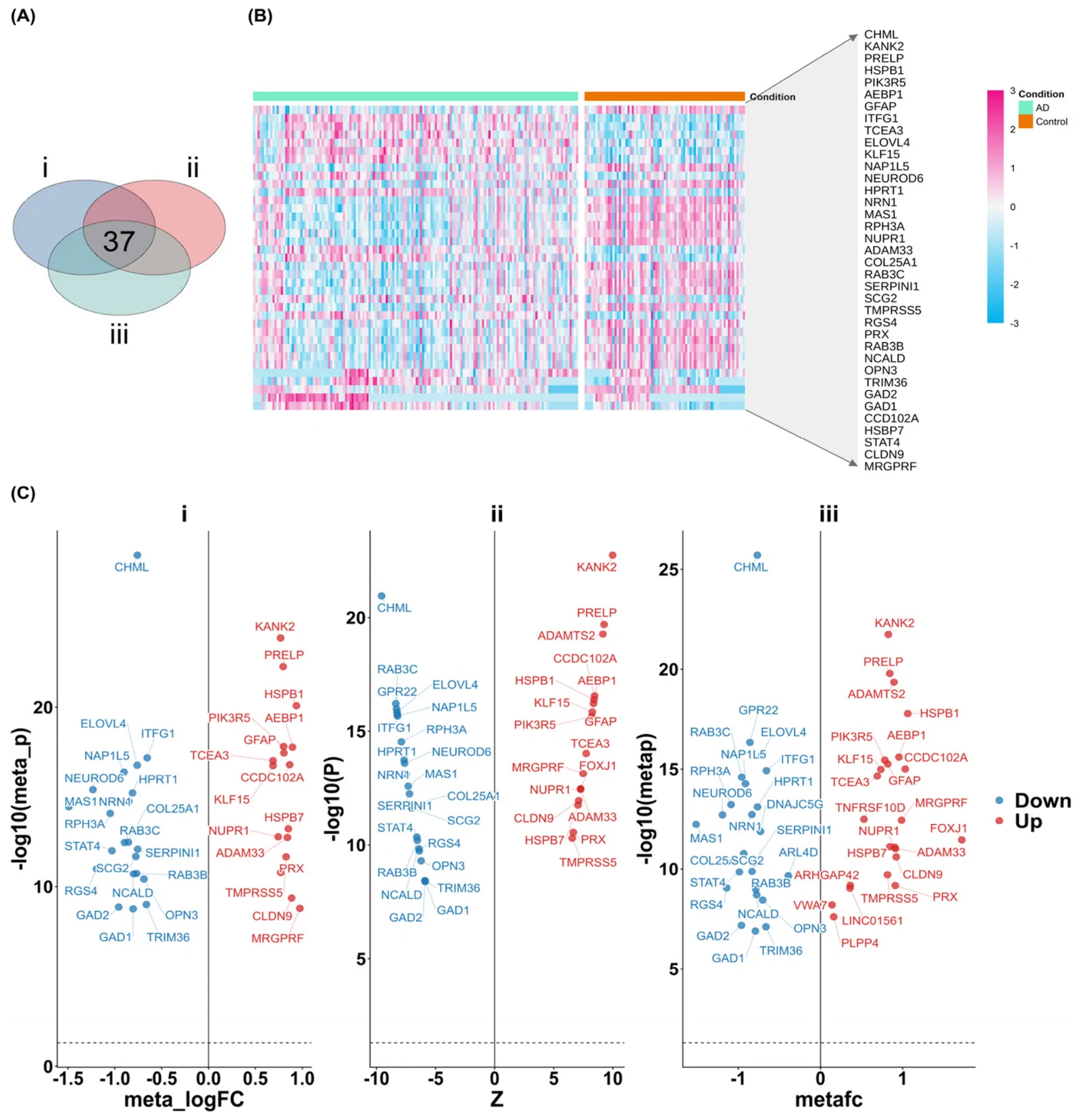
Meta-analysis of differentially expressed genes in Alzheimer’s disease across five transcriptomic datasets. (**A**). Venn diagram showing 37 consensus DEGs identified by three meta-analysis methods: (i) random effects, (ii) Stouffer’s Z, and (iii) MetaVolcano (**B**). Heatmap of ComBat-corrected z-score expression of the 37 DEGs across AD (teal) and control (orange) samples (**C**). Volcano plots from each meta-analysis method: (i). random effects, (ii). Stouffer’s Z, and (iii). MetaVolcano to show the DEGs. Red: upregulated genes, Blue: downregulated genes.

After identifying the DEGs for each individual data, three complementary meta-analytic frameworks: direction-aware Stouffer’s Z-method, DerSimonian–Laird random-effects meta-analysis, and MetaVolcanoR integration, were applied to identify genes that were consistently dysregulated across datasets. After applying the criteria for defining consensus genes as described in the methods, 37 consistently dysregulated genes across different brain regions in AD samples were identified by all three approaches from the 330 genes that entered the meta-analysis (Table S5 and Figure 1A). Under the primary screening criteria, complete leave-one-dataset-out results for all 37 genes are reported in Table S6a. Every gene retained the same direction of effect and remained significant after each dataset omission, with a maximum raw pooled *p* value of 2.0 × 10^−4^. In a separate sensitivity analysis without per-study screening, 14,999 genes entered the meta-analysis; all 37 consensus genes were detected in at least four datasets and remained significant at FDR ≤ 0.05 across all three frameworks and every leave-one-dataset-out analysis (maximum raw pooled *p* = 0.020), although heterogeneity increased, with 19 genes retaining I^2^ < 40% (Table S6b). In a second sensitivity analysis using a per-study FDR ≤ 0.05, no genes in GSE67333 (n = 8) passed the dataset-level screen, limiting pooling to four datasets; only 32 genes entered the meta-analysis, including 11 consensus genes (*CCDC102A*, *CLDN9*, *GFAP*, *ITFG1*, *KANK2*, *KLF15*, *NUPR1*, *PIK3R5*, *PRELP*, *RPH3A*, and *SERPINI1*). All 11 retained their effect direction, remained significant across all three frameworks and every leave-one-dataset-out analysis, and 10 retained I^2^ < 40% (*CLDN9*, I^2^ = 44.2%). The remaining 26 consensus genes were excluded before pooling because they did not achieve dataset-level FDR significance in enough cohorts, rather than because they failed at the meta-analysis stage. Overall, the per-study FDR threshold markedly narrowed gene eligibility, and the retained genes were among those with large pooled effect sizes in primary analysis which continued to show robust cross-study support (Table S6c).

Two additional specifications varied the fold-change threshold while retaining the per-study significance criterion at unadjusted *p* ≤ 0.05 (Table S6c, Panels A, B and C). With a stringent |log_2_FC| > 1.00 threshold, 20 genes entered the meta-analysis, four met the minimum four-dataset requirement, and five consensus genes were retained (*MAS1*, *NEUROD6*, *RGS4*, *RPH3A*, and *HSPB1*). All five preserved their effect direction, showed no detectable heterogeneity (*I*^2^ = 0%), and remained significant across all three meta-analytic frameworks and every leave-one-dataset-out analysis (maximum raw pooled *p* = 2.1 × 10^−4^). With a more permissive |log_2_FC| > 0.25 threshold, 2115 genes entered the meta-analysis and all 37 consensus genes were retained, each with consistent direction, *I*^2^ < 40% (maximum 32.7% for MRGPRF), and significance under every dataset omission (maximum raw pooled *p* = 1.9 × 10^−4^).

As with the FDR specification, all 32 non-retained genes were excluded at the per-dataset screening step because they failed the 2-fold magnitude criterion in enough datasets; none entered the meta-analysis and subsequently lost significance. This indicates that the stricter cutoff primarily reduced eligibility rather than pooled support.

Covariate-adjusted sensitivity models were fitted within each discovery dataset using the available metadata (Table S7a). Age and sex were available in all five datasets, PMI in four, RIN in three, *APOE* genotype in one, and sequencing batch in none. Overall, 175 of 181 estimable gene-by-dataset comparisons (96.7%) retained the direction of effect seen in unadjusted models; no nominally significant gene reversed direction, and all six discordant estimates were non-significant in both models (*p* > 0.24). All 37 genes remained nominally significant after adjustment in at least one dataset, 25 in three or more, and *KANK2* in all five.

Adjustment generally reduced effect sizes and nominal significance, particularly in smaller or structurally imbalanced cohorts. In GSE95587, the largest and most demographically balanced dataset, all 37 genes remained directionally concordant and 35 of 36 retained significance. Greater attenuation occurred in GSE159699 and GSE203206, where age was closely linked to case–control structure and adjustment likely absorbed part of the diagnosis signal. GSE67333 (n = 8; 3 residual df) was too small for stable estimation, with four genes not estimable, and is reported for completeness only (Table S7a,b).

The same pattern persisted under stricter criteria (Table S7a). At FDR < 0.05, 79 comparisons were significant before adjustment and 56 after, with no new significant findings introduced by adjustment. GSE67333 and GSE203206 had no FDR-significant comparisons in either model, consistent with their limited within-study power. Adding |log_2_FC| > 0.58 reduced counts from 134 to 70 at nominal significance and from 69 to 32 at FDR, largely reflecting smaller effect sizes: the median |log_2_FC| fell from 0.727 to 0.583. This was most evident in GSE95587, where 34 of 36 genes remained FDR-significant, but only 21 also exceeded |log_2_FC| > 0.58.

Using a per-study FDR ≤ 0.05 in place of the unadjusted *p*-value threshold markedly reduced the number of genes eligible for meta-analysis. Only 32 genes met the minimum dataset requirement, including 11 of the 37 consensus genes: CCDC102A, CLDN9, GFAP, ITFG1, KANK2, KLF15, NUPR1, PIK3R5, PRELP, RPH3A, and SERPINI1. All 11 remained significant across the three meta-analytic frameworks, showed the same direction of effect as in the primary analysis, and remained significant in every leave-one-dataset-out analysis. Ten of the 11 also retained low-to-moderate heterogeneity (*I*^2^ < 40%), with CLDN9 showing slightly higher heterogeneity (*I*^2^ = 44.2%).

The remaining 26 consensus genes were not excluded because of loss of significance at the meta-analysis stage; rather, they failed to pass the more stringent per-study FDR screen in enough datasets to satisfy the minimum dataset requirement for pooling. Notably, the 11 genes retained under this specification were among those with the largest pooled effect sizes in the primary analysis.

Among the consensus genes, 16 were consistently upregulated, and 21 were downregulated across all datasets (Figure 1B). The meta-analytic effect sizes and significance of each gene from each method are displayed in the corresponding volcano plots (Figure 1C). The upregulated gene set included *KANK2* (a therapeutic target for tauopathies) [28], *GFAP* and *HSPB1* (astrocytic biomarkers) [21,29,30], *AEBP1* and *PRELP* (ECM components), and *ADAM33* (a matrix-modifying enzyme) [31,32], indicating major remodeling of the brain’s ECM and a shift toward a proinflammatory and profibrotic environment. Conversely, the downregulated genes are characterized by synaptic-associated factors, including *RAB3B*, *RAB3C*, *RPH3A*, and *SCG2*, alongside *GAD1* and *GAD2*, which are central to GABAergic neurotransmission [33,34,35]. Other notably downregulated features include *NEUROD6*, *NRN1*, and *NCALD*, suggesting broad suppression of the neuronal and synaptic signaling machinery [36,37,38].

**Figure 2:**
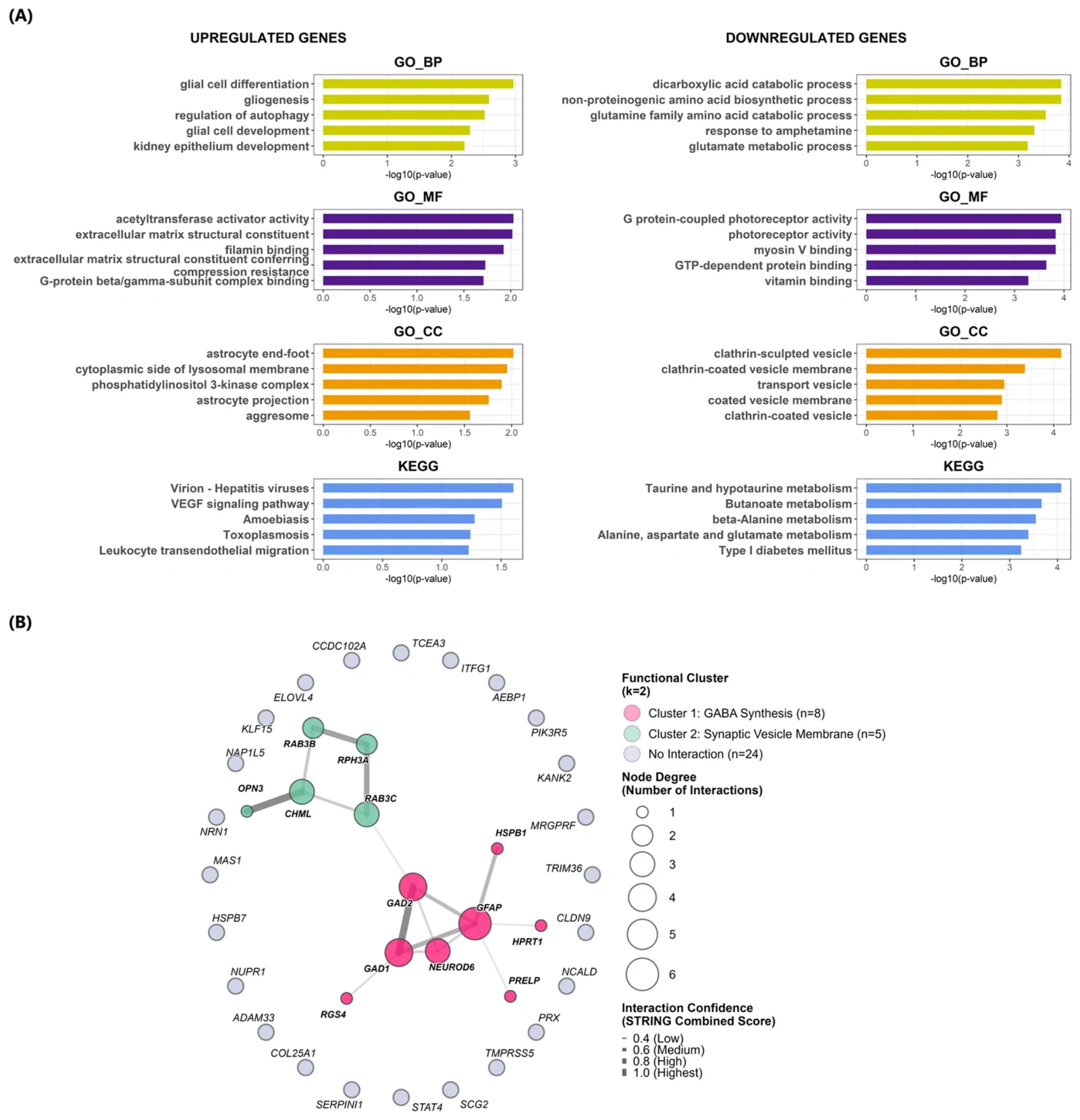
Functional enrichment of the dysregulated consensus genes across different AD brain regions. (**A**). Gene Ontology (GO) Biological Process (BP) (yellow), Molecular Function (MF) (purple), Cellular Component (CC) (orange), and KEGG (blue) pathway enrichment analyses were performed separately for upregulated (on left) and downregulated (on right) consensus genes. (**B**). STRING protein–protein interaction network of the 37 consensus DEGs. Functional clustering (k = 2) identified two modules: GABA synthesis (pink, n = 8) and synaptic vesicle membrane (green, n = 5). Node size indicates interaction degree; edge thickness reflects confidence score.

Pathway enrichment analysis of the 37 consensus genes showed that the upregulated genes clustered in glial/ECM- and lysosome-associated processes, whereas the downregulated genes were enriched in the synaptic vesicle, neurotransmitter receptor, and ion transport pathways (Figure 2A). This finding reinforces a pattern of increased glial and ECM gene expression along with synaptic and neuronal dysfunction in AD, reflecting the results of the individual data analyses. Furthermore, PPI network analysis revealed a small but highly connected network (37 nodes, 16 edges; expected edges = 3; PPI enrichment *p* = 4.64 × 10^−7^; Figure 2B). K-means clustering (k = 2) identified two distinct modules: one enriched for GABA synthesis, taurine/hypotaurine metabolism, and neurotransmitter biosynthesis and another focused on anchored synaptic vesicle membrane components. These findings point to a coordinated disruption of both neurotransmitter metabolism and synaptic vesicle organization in AD.

### 3.4. Association of Consensus Genes with Braak Stage

Among the 201 samples with available Braak stage information, all 16 upregulated consensus genes were positively correlated with Braak stage, while all 21 downregulated genes showed negative correlations. After FDR correction, 36 of the 37 correlations remained significant (FDR ≤ 0.05), with *HSPB7* as the only exception. The strongest positive correlations were observed for *KANK2* (ρ = 0.449), *KLF15* (ρ = 0.385), and *PRX* (ρ = 0.361), while the strongest negative correlations were observed for *RPH3A* (ρ = −0.485), *NRN1* (ρ = −0.464), and *NEUROD6* (ρ = −0.455) (Figure S2).

**Figure 3:**
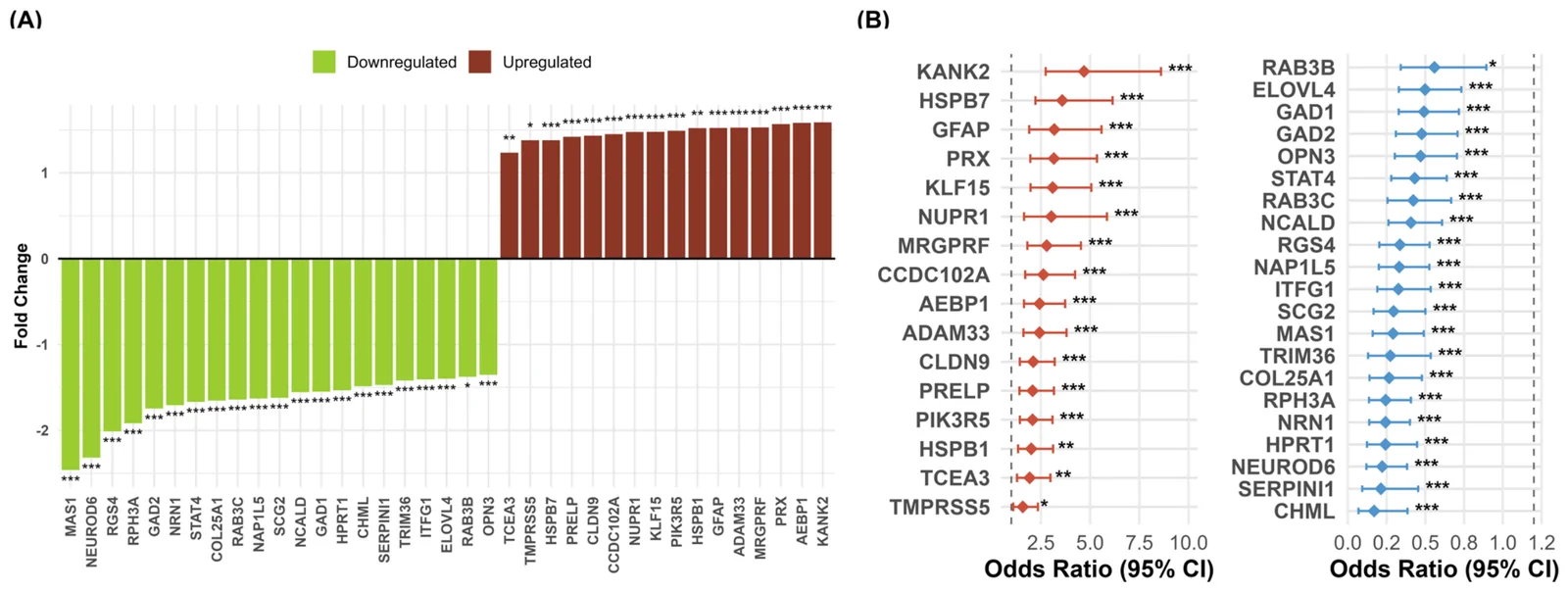
Association of consensus gene expression with Braak stage across all brain region. (**A**) Waterfall plot showing the fold change in ComBat-corrected, z-score normalized gene expression between Low (Braak ≤ 3) and High (Braak > 3) groups, pooled across all brain regions. Dark red bars indicate genes upregulated in the High Braak group; green bars indicate genes downregulated in the High Braak group. Genes are ordered by ascending fold change. Asterisks denote statistical significance from ANCOVA adjusting for brain region, age, and sex (* FDR ≤ 0.05, ** FDR ≤ 0.01, *** FDR ≤ 0.001). (**B**) Forest plots of odds ratios (ORs) from logistic regression testing the probability of High Braak classification per unit increase in z-score normalized gene expression, adjusting for brain region, age, and sex. Left panel (red): upregulated genes with OR > 1; right panel (blue): downregulated genes with OR < 1. Diamonds represent point estimates; horizontal lines indicate 95% confidence intervals. Dashed vertical lines mark OR = 1 (null). * FDR ≤ 0.05, ** FDR ≤ 0.01, *** FDR ≤ 0.001.

Analysis comparing the high (Braak > 3) and low (Braak ≤ 3) Braak groups confirmed that all 37 consensus genes were significantly differentially expressed between the groups after adjusting for brain region, age, and sex (FDR ≤ 0.05; Figure 3A). Downregulated genes, such as *MAS1*, *NEUROD6*, *RGS4*, and *RPH3A*, presented the largest fold decreases in the high Braak group, whereas the upregulated genes, including *KANK2*, *AEBP1*, *PRX*, and *GFAP* showed the highest increased expressions in AD.

Logistic regression further demonstrated that all 37 genes were significantly associated with Braak classification (FDR ≤ 0.05; Figure 3B). *KANK2* showed the highest odds ratio (OR) among the upregulated genes (OR = 4.69; 95% CI: 2.74–8.60; FDR < 0.001), followed by *HSPB7* (OR = 3.58, 95% CI: 2.22–6.14) and *GFAP* (OR = 3.18, 95% CI: 1.91–5.58), indicating that increased expression of these genes was associated with increased odds of advanced neuropathology. Among the downregulated genes, the strongest protective associations were observed for *CHML* (OR = 0.17, 95% CI: 0.07–0.39), *SERPINI1* (OR = 0.21, 95% CI: 0.09–0.45), and *NEUROD6* (OR = 0.22, 95% CI: 0.12–0.38), indicating that higher expression of these genes was associated with lower odds of advanced AD stages.

**Figure 4:**
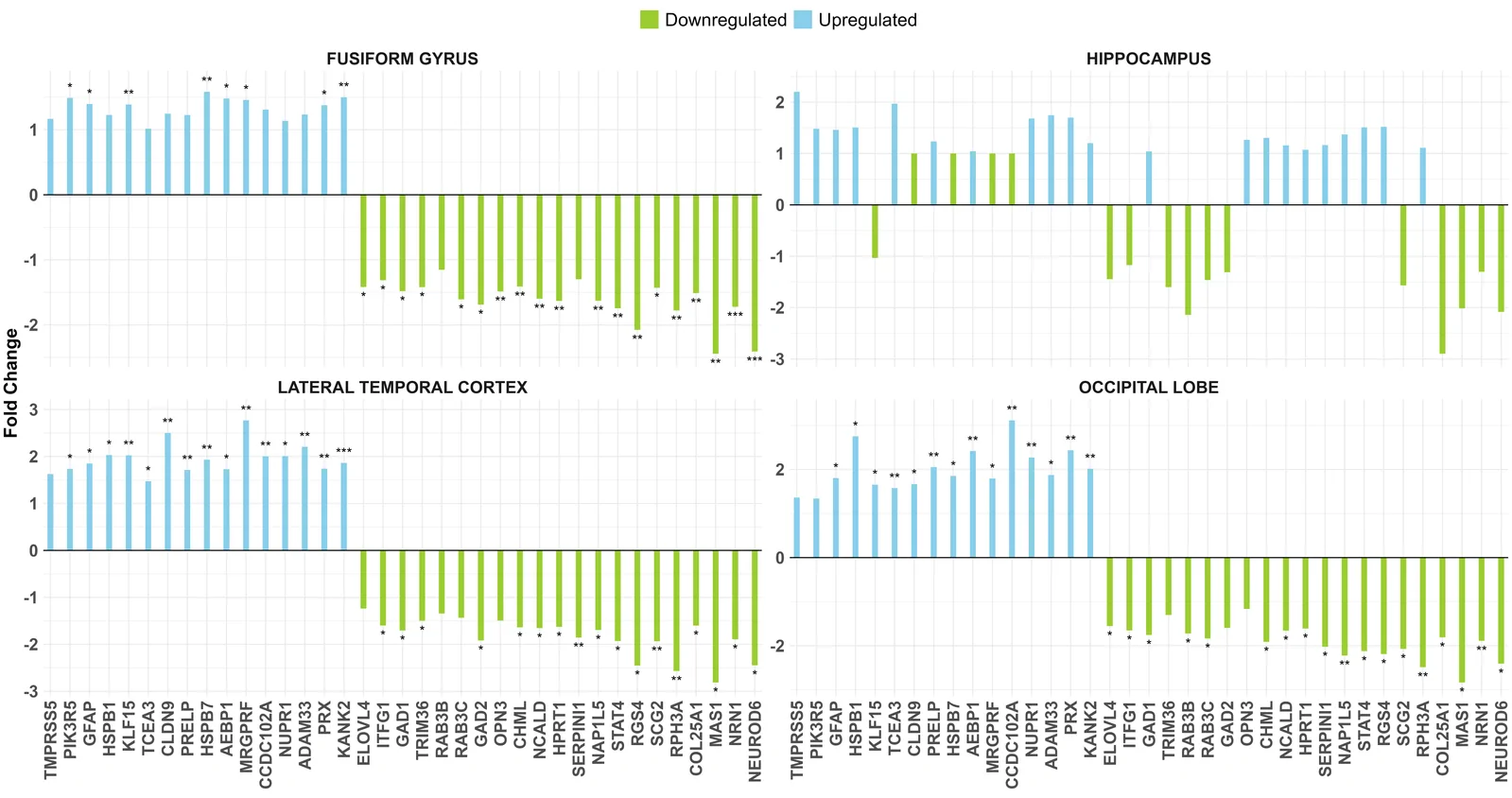
Region-stratified gene expression differences between High and Low Braak groups. Waterfall plots showing the fold change in ComBat-corrected, z-score normalized gene expression between Low (Braak ≤ 3) and High (Braak > 3) groups for each of the 37 consensus genes, stratified by brain region (fusiform gyrus, hippocampus, lateral temporal cortex, and occipital lobe). Blue bars indicate genes upregulated in the High Braak group; green bars indicate genes downregulated. Genes are ordered by overall mean fold change across all regions. Asterisks denote statistical significance from per-region ANCOVA adjusting for age and sex (* FDR ≤ 0.05, ** FDR ≤ 0.01, *** FDR ≤ 0.001).

Region-stratified analysis, after adjusting for age and gender, revealed that the lateral temporal cortex and occipital lobe showed the most widespread associations with the BRAAK stage, with 32 of 37 genes reaching significance (FDR ≤ 0.05) in each region. The fusiform gyrus, followed with 27 of 37 genes, was significantly associated with the BRAAK stage. While the hippocampus showed consistent directional trends, no genes reached statistical significance, likely due to the limited sample size in this subgroup (Figure 4).

### 3.5. Validation Performance of the Identified Gene Signature in Independent Cohorts

**Figure 5:**
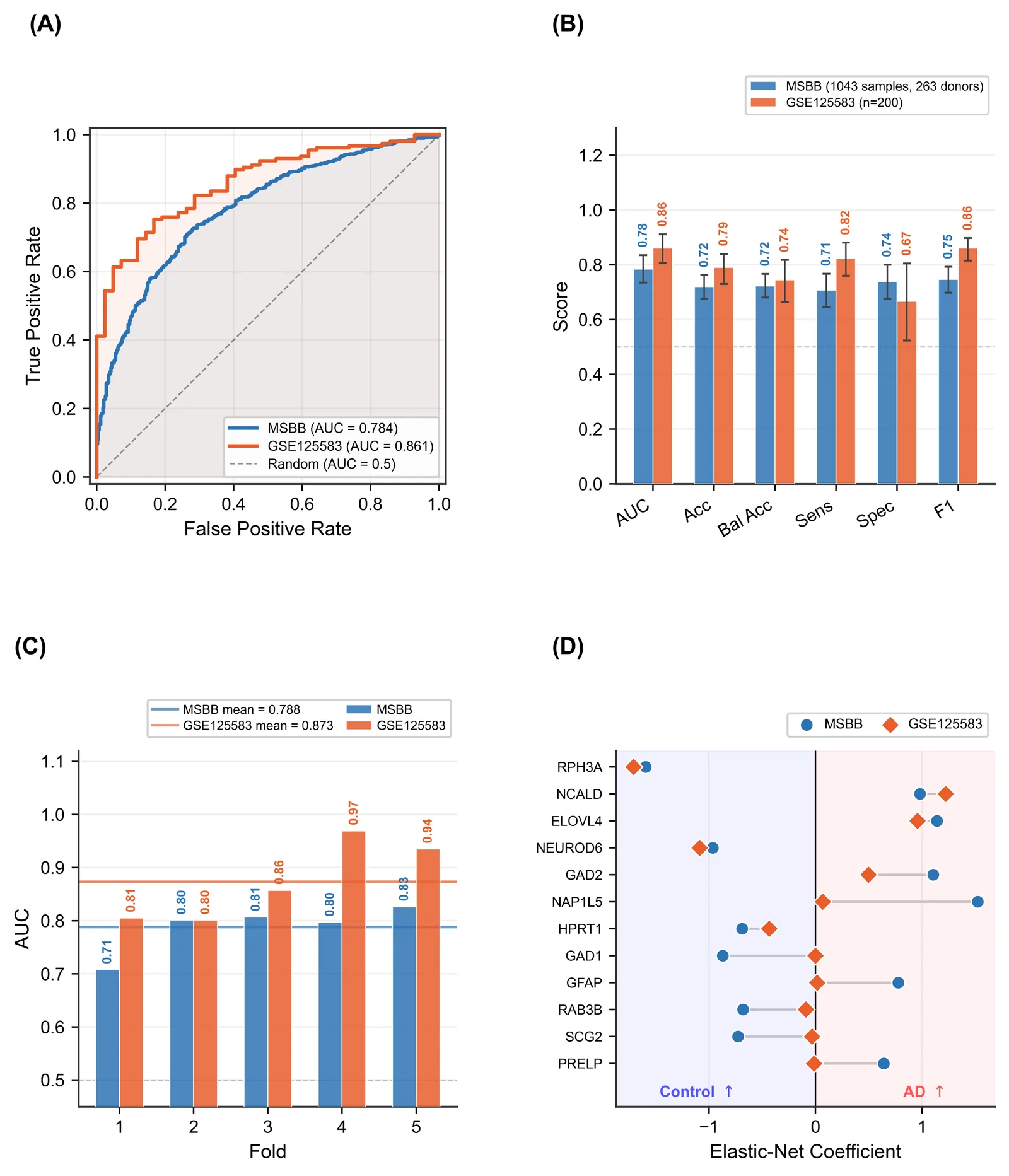
Validation of the 37-gene signature in two independent cohorts. (**A**) Receiver operating characteristic (ROC) curves for MSBB (1043 samples from 263 donors) and GSE125583 (n = 200) using stratified 5-fold cross-validation. In MSBB, samples were grouped by donor during cross-validation, whereas sample-level stratification was used for GSE125583 because each donor contributed a single sample. The dashed diagonal represents random classification (AUC = 0.5). (**B**) Performance metrics for both cohorts, with 95% confidence intervals estimated from 1000 bootstrap replicates at the donor level for MSBB and the sample level for GSE125583. (**C**) AUC values across the five folds, with horizontal lines showing the mean AUC for each cohort (MSBB: 0.788; GSE125583: 0.873). The greater variability across folds in GSE125583 likely reflects the smaller number of control samples in each fold. (**D**) Elastic-net logistic regression coefficients for the 15 genes with the largest contributions in each cohort. Positive coefficients indicate an association with AD, whereas negative coefficients indicate an association with control status. All analyses used an elastic-net logistic regression model (L1 ratio = 0.5, C = 1.0) with balanced class weighting.

In order to validate the identified genes as consistent biomarkers for AD prediction, we further tested their predictive power using two independent datasets: MSBB (n = 1043 samples from 263 donors; 607 AD, and 436 controls) and GSE125583 (n = 200; 158 AD, and 42 controls). All 37 identified genes were available in MSBB, and 36 were found in GSE125583. Using donor-grouped, stratified 5-fold cross-validation, the signature achieved an AUC of 0.784 (95% CI: 0.735–0.835) in MSBB and 0.861 (95% CI: 0.806–0.912) in GSE125583 (Table 2; Figure 5A,B). Performance across folds was relatively consistent in MSBB (AUC: 0.708–0.826), whereas greater variability was observed in GSE125583 (AUC: 0.805–0.969), likely due to the smaller number of control samples within each fold (Figure 5C). Similar results were obtained using leave-one-donor-out cross-validation, with AUCs of 0.796 (95% CI: 0.747–0.844) in MSBB and 0.888 (95% CI: 0.836–0.936) in GSE125583. Complete performance metrics and corresponding confidence intervals are reported in Table 2.

Comparison of the coefficients of the Elastic-Net models across the two cohorts revealed that *RPH3A*, *NCALD*, *ELOVL4*, and *NEUROD6* were among the top contributors in both datasets and retained the same direction of effect (Figure 5D). Cohort-specific differences were also observed: *NAP1L5* was the top-ranked gene in MSBB but contributed minimally in GSE125583, where *NRN1* emerged as the strongest feature. Coefficient estimates differed across cohorts, as expected for elastic-net models when predictors are correlated. For example, genes such as *GAD1/GAD2* and *RAB3B/RAB3C* may share predictive information, allowing their relative weights to vary between cohorts.

## 4. Discussion

In this study, we used a novel multicohort meta-analytic approach to analyze five datasets across different brain regions ranging from the hippocampus to the cortex, capturing the spatial distribution of AD pathology across regions affected at different disease stages. This identified 37 consensus dysregulated genes consistently altered across the brain. Further analysis showed that these genes are involved in synaptic vesicle organization, neurotransmitter receptor signaling, and glial-mediated inflammatory pathways and are highly associated with Braak stages. Validation across two independent datasets demonstrated that this gene signature achieves high predictive accuracy for AD, with AUCs of 0.78 and 0.86, respectively.

The five RNA-seq datasets included 154 AD and 76 healthy control (HC) samples. Comparing AD to HC, individual data analysis identified a varying number of DEGs. Despite substantial heterogeneity in brain regions [39], sequencing depth, and cohort composition, differential expression analyses of individual data consistently revealed two dominant patterns in AD samples: (i) upregulation of immune-, glial-, and stress-associated genes and (ii) downregulation of genes involved in synaptic transmission, neurotransmitter metabolism, and neuronal structure [40,41,42]. These patterns were observed across cortical and hippocampal regions, suggesting that they represent core features of AD-related neurodegeneration rather than region-specific phenomena.

AD is characterized by selective regional vulnerability, where the staggered accumulation of pathology across the brain correlates with advancing Braak stages, reflecting the characteristic spatial distribution of neurodegeneration [43,44,45]. To enhance the robustness of our biomarker discovery and identify shared signatures across heterogeneous brain regions, we utilized an integrative meta-analytic framework, incorporating direction-aware Stouffer’s Z-scores, random effects modeling, and MetaVolcanoR analysis, and identified 37 consensus dysregulated genes across diverse brain regions, which showed minimal inter-study heterogeneity under primary screening criteria. Expression of 36 of the 37 genes was significantly associated with Braak stage, with upregulated genes showing positive correlations and downregulated genes showing negative correlations.

Pathway enrichment analysis of the 37 consensus genes revealed a clear dichotomy: upregulation of glial, immune, (ECM), and stress response programs alongside the suppression of neuronal and synaptic genes, which is consistent with individual data analyses. This pattern aligns with the prevailing model of AD pathogenesis, where reactive astrogliosis and neuroinflammation accompany progressive synaptic loss and neuronal dysfunction [46]. Notably, this transcriptional signature is consistent with a broader framework of disrupted glial-synapse iterations at the tripartite synapse, as reactive glia shift from homeostatic support to maladaptive, inflammatory phenotypes [40,47,48]. This may include the aberrant reactivation of complement-dependent pruning, which drives synapse loss and cognitive decline [49]. The convergence of these patterns across anatomically and temporally distinct circuits indicates that our gene signature reflects core features of AD-related neurodegeneration rather than region-specific idiosyncrasies.

PPI network analysis further confirmed these results. Two principal functional modules were identified: one focused on neurotransmitter biosynthesis (e.g., *GAD1*, *GAD2*, *GFAP*, *NEUROD6*, *RGS4*, *HSPB1*, *HPRT1*, and *PRELP*), and the other focused on synaptic vesicle membrane components (e.g., *RAB3B*, *RAB3C*, *RPH3A*, *CHML*, and *OPN3*) [36]. Together, these analyses suggest that AD pathology arises from the coordinated disruption of interconnected hubs linking ECM remodeling and cytoskeletal integrity to a central collapse of GABAergic neurotransmission and vesicle dynamics.

Among the 37 genes, the upregulation of astrocytic markers such as *GFAP* and *HSPB1* corroborates extensive immunohistochemical and single-nucleus RNA-seq evidence demonstrating pronounced astrocyte reactivity in proximity to amyloid plaques and neurofibrillary tangles [21,29,30]. Similarly, elevated expression of *KANK2*, ECM components (*AEBP1* and *PRELP*), and matrix-modifying enzyme (*ADAM33*) suggests active tissue remodeling, potentially contributing to aberrant neuronal connectivity and impaired synaptic plasticity [31,32].

Conversely, the downregulation of synaptic vesicle-associated genes (*RAB3B*, *RAB3C*, *RPH3A*, and *SCG2*) and the GABAergic neurotransmission machinery (*GAD1* and *GAD2*) reflects a well-documented synaptic pathology that strongly correlates with cognitive decline in AD [33,34,35]. This consistent suppression across the hippocampus, the fusiform gyrus, the lateral temporal cortex, and the occipital lobe underscore a pervasive disruption of synaptic integrity. Specifically, the downregulation of *NEUROD6*, a transcription factor critical for neuronal differentiation and survival [36], vesicle trafficking regulators (*NCALD*, *NRN1*), and calcium-dependent neurotransmitter release (*RAB3* isoforms) implicates impaired synaptic vesicle dynamics as a central feature of neurodegeneration [34,50]. Furthermore, the widespread deficit in *GAD1*/2 suggests that GABAergic inhibitory neurotransmission may exacerbate excitotoxicity and network hyperexcitability in AD [33]. Together, these findings suggest that losing an inhibitory tone and vesicle-mediated signaling reduces synaptic density, driving cognitive decline. Consequently, targeting GABAergic signaling or RAB-mediated vesicle dynamics may be effective therapeutic strategies for AD [51].

A key finding of this study is the strong correlation between dysregulated consensus gene expression and Braak stages, a neuropathological measure of neurofibrillary tangle burden and disease progression [52]. These changes are consistent with responses to tau aggregation and with downstream neuronal dysfunction although our cross-sectional design cannot establish this. The upregulation of stress-responsive genes such as *HSPB1* may represent compensatory cellular stress responses to proteotoxic tau species [53], and, the lower expression of synaptic genes observed at higher Braak stages is consistent with the anteroposterior spread of tau pathology and the corresponding loss of synaptic density observed in postmortem studies [54]. These associations should nonetheless be interpreted cautiously. Braak stage is itself closely correlated with diagnosis and with the overall extent of neurodegeneration, and the consensus genes were selected on the basis of case–control differential expression, so associations with stage partly recapitulate the criterion by which the genes were identified. We therefore cannot determine whether they reflect stage-specific mechanisms or the presence and severity of AD-related neurodegeneration more generally; resolving this would require cohorts in which stage varies within diagnostic groups, particularly at intermediate stages, which our exclusion of mild cognitive impairment removed. Because all data are cross-sectional and post-mortem, differences between individuals at different Braak stages cannot be interpreted as within-individual progression. These genes therefore represent candidate molecular correlates of neuropathological stage rather than established markers of disease progression in postmortem tissue.

Several consensus genes identified in our analysis have been implicated in AD pathogenesis through independent lines of evidence, lending biological plausibility to our findings. *KANK2*, one of the most upregulated genes in AD in our study, has been suggested as a therapeutic target for tauopathy [28]. *GFAP*, a canonical marker of astrocyte activation, has been extensively studied as a neuroinflammatory indicator in AD and is currently being evaluated as a blood-based biomarker for disease monitoring [55]. The heat shock proteins HSPB1 and HSPB7 are molecular chaperones that modulate protein aggregation and cellular stress responses, and their upregulation may reflect cellular attempts to mitigate the proteotoxic stress induced by amyloid-beta and tau accumulation [29]. Pharmacologically targeting these stress response pathways may offer therapeutic opportunities to enhance neuronal resilience in the context of AD-related proteostasis dysfunction. In addition, our identification of previously understudied genes in AD, such as *ELOVL4*, *OPN3*, *KLF15*, and *PRX*, highlights novel molecular candidates that may contribute to disease pathogenesis and warrant further functional investigation.

The 37-gene signature showed consistent classification performance in two independent AD brain transcriptomic cohorts, achieving AUCs of 0.784 in MSBB and 0.861 in GSE125583 under stratified five-fold cross-validation. Similar performance was observed with leave-one-donor-out analysis. The higher AUC in GSE125583 may partly reflect its smaller sample size and greater class imbalance (79% AD). However, balanced accuracy remained above 0.74, suggesting that the observed performance was not simply driven by the higher proportion of AD samples.

A core subset of genes, *RPH3A*, *NCALD*, *ELOVL4*, and *NEUROD6,* consistently ranked among the top features in both cohorts with conserved directionality, implicating disruptions in synaptic vesicle trafficking, calcium signaling, and lipid metabolism in AD. *GFAP* also contributed positively to AD in both datasets, suggesting that the signature captures glial and neuronal expression changes. Cohort-specific differences, such as the prominence of *NAP1L5* in MSBB and *NRN1* in GSE125583, may reflect biological heterogeneity or technical variation between datasets.

Cross-referencing the 37 consensus genes against published human AD single-nucleus RNA-seq data (Table S8) identified 16 genes reported as cell-type-specific DEGs, comprising 2 upregulated (*GFAP*, *HSPB1*) and 14 downregulated genes. Thirteen of these were concordant in direction with our bulk results, with downregulated genes mapping predominantly to excitatory and inhibitory neurons and upregulated genes to astrocytes. Only three genes (*NCALD*, *RPH3A*, *SERPINI1*) showed cell-type-dependent directionality, with *SERPINI1* reduced in excitatory neurons but increased in oligodendrocytes [56].

While this meta-analysis offers several advantages, some limitations remain. First, individual dataset sizes were modest (8–117 subjects). Precision weighting limits the influence of the smallest cohorts, which contribute approximately 9% of the pooled weight compared with 35% for the largest, and consensus genes were required to show consistent effects in at least four of five datasets, so no single cohort can drive inclusion. Effect estimates from the smallest cohorts are nonetheless likely inflated in magnitude, and with only five studies the between-study variance component is estimated imprecisely, so heterogeneity statistics should be regarded as approximate [57]. Heterogeneity estimates were sensitive to gene filtering, with approximately half of the consensus genes exceeding the I^2^ threshold when gene filtering was not applied. This finding suggests that the primary analysis may underestimate between-study variability in effect sizes for some genes.

The eligible gene pool was sensitive to the choice of per-study screening threshold, with an FDR-based criterion reducing the number of genes entering the meta-analysis and retaining only 11 of the 37 consensus genes. Accordingly, the consensus signature should be interpreted as reflecting consistent cross-study evidence under the primary screening strategy, rather than requiring within-study FDR significance in every contributing cohort.

Second, using exclusively postmortem tissue may capture end-stage pathology rather than the earliest transcriptomic changes. Third, because our analysis is based on bulk RNA-seq data, we cannot determine whether the observed expression changes reflect differences in cell-type composition, changes within individual cell types, or a combination of both. Comparison with published single-nucleus data supports the expected cell-type associations (Table S8), but separating these effects would require matched single-nucleus data. Fourth, the primary models were unadjusted because covariate availability differed across datasets. In dataset-specific sensitivity models, 96.7% of estimates retained the same direction and no significant effect reversed sign, although significance was reduced in some cohorts, particularly where age was confounded with case–control structure (Table S7a,b). Because sequencing batch and other factors such as comorbidities and medication use were unavailable, residual confounding cannot be excluded. Finally, although the 37-gene signature was evaluated in independent cohorts, model coefficients were estimated separately within each cohort. Thus, these analyses demonstrate the discriminative value of the predefined gene set rather than external validation of a fully trained classifier. Future studies using cross-cohort train–test designs are needed to further establish its generalizability.

## 5. Conclusions

In summary, our integrated cross-regional meta-analysis identified a robust transcriptional signature of AD defined by increased glial, reduced synaptic gene expression, and a strong association with neuropathological severity. By integrating multiple meta-analytic strategies with network-level analysis and pathological staging, our study provides a comprehensive framework for identifying reproducible molecular features of neurodegeneration. These findings offer a refined set of candidate genes to guide future mechanistic studies and biomarker development.

## Figures and Tables

**Table 1:** Summary of RNA-seq datasets used for the study.

Datasets		Total Subjects	Tissue/Brain Region	DEGs Up/Down	AD (M/F)	Control (M/F)	Age (Mean ± SD)	Braak Stage
**Testing** **datasets**	GSE159699	30	Lateral Temporal Cortex	869/316	11/1	16/2	63.8 ± 9.5	Yes
	GSE67333	8	Hippocampus	326/261	1/3	2/2	82.7 ± 3.0	Yes
	GSE203206	47	Occipital Lobe	753/941	23/16	4/4	74.1 ± 13.4	Yes
	GSE95587	117	Fusiform gyrus	459/746	42/42	23/10	85.5 ± 7.5	Yes
	GSE278723	28	CA1	2259/1857	6/9	8/5	82.5 ± 9.2	No
			CA2	1954/2023				
			CA3	2044/1815				
			CA4	1594/1286				
			Entorhinal cortex	2163/1881				
**Validation datasets**	MSBB	1043 samples (263 donors)	superior temporal gyrus, frontal pole, parahippocampal gyrus, prefrontal cortex, inferior frontal gyrus		226/381	183/253	82.6 ± 7.9	Yes
	GSE125583	200	Fusiform gyrus		85/71 *	19/23	84.0 ± 7.9	Yes

Note: CA: cornu ammonis, DEGs: Differentially expressed genes, F: Female, M: Male, SD: Standard Deviation. * Age & Gender is not available for one donor. For MSBB, counts refer to tissue samples and donors contribute to multiple brain regions are considered once per sample.

**Table 2:** Validation performance of the 37-gene signature across two independent cohorts.

Cohort/Method	AUC	Accuracy	Bal. Acc.	Sensitivity	Specificity	Precision	F1
**MSBB** **—donor-grouped 5-fold CV**	0.784 (0.735–0.835)	0.720 (0.677–0.763)	0.723 (0.681–0.767)	0.707 (0.645–0.767)	0.739 (0.676–0.801)	0.790 (0.729–0.848)	0.746 (0.698–0.793)
**MSBB—leave-one-donor-out CV**	0.796 (0.747–0.844)	0.722 (0.681–0.764)	0.725 (0.681–0.767)	0.708 (0.650–0.768)	0.741 (0.674–0.803)	0.792 (0.731–0.853)	0.748 (0.702–0.794)
**GSE125583—5-fold CV**	0.861 (0.806–0.912)	0.790 (0.730–0.840)	0.745 (0.664–0.818)	0.823 (0.760–0.882)	0.667 (0.524–0.805)	0.903 (0.853–0.946)	0.861 (0.815–0.898)
**GSE125583—LOOCV**	0.888 (0.836–0.936)	0.835 (0.785–0.885)	0.808 (0.737–0.878)	0.854 (0.802–0.911)	0.762 (0.630–0.889)	0.931 (0.892–0.971)	0.891 (0.854–0.927)

Note: Values are point estimates with 95% confidence intervals from bootstrap resampling. Abbreviations: AUC, area under the receiver operating characteristic curve; Bal. Acc., balanced accuracy; CV, cross validation; LOOCV, Leave-one-out cross validation; MSBB, Mount Sinai Brain Bank.

## Data Availability

The data that support the findings of this study are openly available in the GEO database and ADKP (MSBB data (syn27068756)): https://adknowledgeportal.synapse.org/, accessed on 19 August 2026. Analysis code and reproducibility materials are publicly available through the project GitHub repository (https://github.com/Code-krafter22/meta-analysis_AD_biomarker, accessed on 6 September 2026). The exact code version corresponding to the submitted manuscript is preserved as GitHub Release v1.1.0 (https://github.com/Code-krafter22/meta-analysis_AD_biomarker/releases/tag/v.1.1.0, accessed on 2 September 2026 ).

## Supplementary Materials

The following supporting information can be downloaded at: https://www.mdpi.com/article/10.3390/biomedicines14092032/s1. Figure S1: Differential expression across multiple brain regions; Figure S2: Spearman correlations between consensus gene expression and Braak stage; Table S1: Demographic characteristics and availability of clinical and technical metadata across the five discovery datasets; Table S2: Differentially expressed genes in each discovery dataset: (a) GSE159699 (b) GSE67333 (c) GSE203206 (d) GSE95587 (e) GSE278723; Table S3: Heterogeneity statistics for differentially expressed genes in GSE278723; Table S4: Functional enrichment and pathway analysis; Table S5: Top significantly differentially expressed genes identified by all three meta-analysis methods; Table S6: Sensitivity of the consensus signature to gene-selection criteria: (a) complete leave-one-dataset-out sensitivity analysis for the 37 consensus genes, (b) meta-analysis results for the 37 consensus genes without per-study significance filtering, (c) screening-sensitivity analyses, comprising a comparison of specifications (Panel (A)), per-gene results under the per-study FDR specification (Panel (B)), and per-gene results under the fold-change threshold specifications (Panel (C)); Table S7: Covariate-adjusted sensitivity analysis: (a) results by dataset, (b) results by gene; and Table S8: Concordance of the 37 consensus genes with cell-type-specific differential expression in AD single-nucleus RNA-seq data.

## Abbreviations

The following abbreviations are used in this manuscript:

Aβ	Amyloid-β
AD	Alzheimer’s disease
ADKP	Alzheimer’s disease knowledge portal
ANCOVA	Analysis of covariance
ANOVA	Analysis of variance
APOE	Apolipoprotein E
AUC	Area under the curve
Bal. Acc.	Balanced accuracy
BP	Biological process
CA	Cornu ammonis
CC	Cellular component
CI	Confidence interval
CPM	Counts per million
CV	Cross validation
DE	Differential expression
DEGs	Differentially expressed genes
ECM	Extracellular matrix
FDR	False discovery rate
GABA	Gamma-aminobutyric acid
GEO	Gene expression omnibus
GO	Gene ontology
HC	Healthy control
HGNC	Hugo gene nomenclature committee
KEGG	Kyoto encyclopedia of gene and genomes
LOOCV	Leave one out cross validation
LogFC	Log fold change
MCI	Mild cognitive impairment
MF	Molecular function
MSBB	Mount Sinai brain bank
OR	Odds ratio
PPI	Protein-protein interaction
RNA-seq	RNA sequencing
ROC	Receiver operating characteristic
SD	Standard deviation
SE	Standard error
TMM	Trimmed mean of m-values
