# Supplementary material for: A Cross-Region Meta-Analysis and Machine Learning Identifies a 37-Gene Signature Associated with Alzheimer’s Disease
Source: Biomedicines. 2026 Sep 10;14(9):2032. doi: 10.3390/biomedicines14092032 (PMC13604570; doi:10.3390/biomedicines14092032)
Supplement: Supplementary file 1 [file biomedicines-14-02032-s001.zip › Supplementary Figures.pdf]

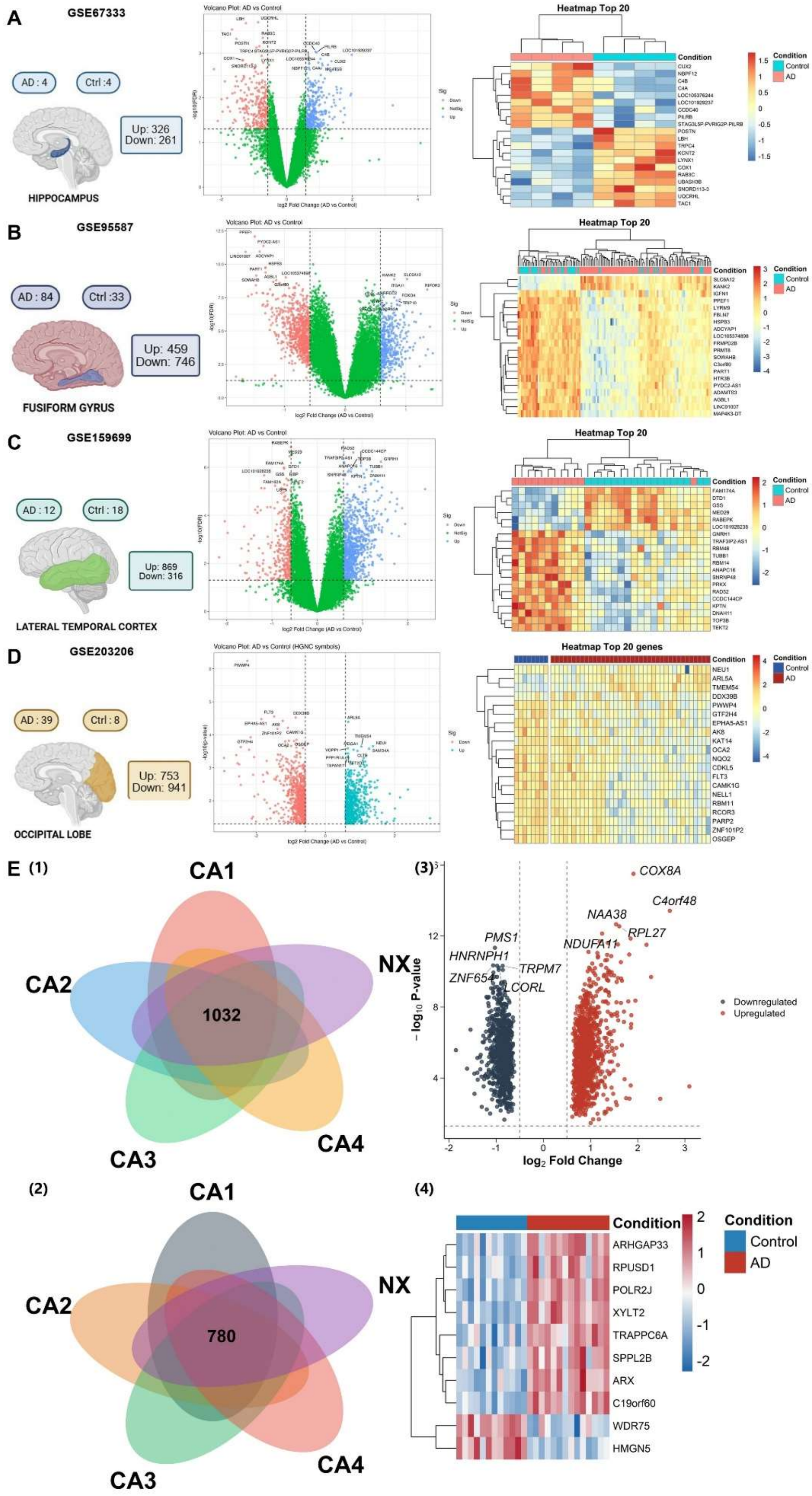

A
GSE67333
AD : 4
Ctrl :4
Up: 326
Down: 261
HIPPOCAMPUS
Volcano Plot: AD vs Control
Heatmap Top 20
B
GSE95587
AD : 84
Ctrl :33
Up: 459
Down: 746
FUSIFORM GYRUS
Volcano Plot: AD vs Control
Heatmap Top 20
C
GSE159699
AD : 12
Ctrl : 18
Up: 869
Down: 316
LATERAL TEMPORAL CORTEX
Volcano Plot: AD vs Control
Heatmap Top 20
D
GSE203206
AD : 39
Ctrl : 8
Up: 753
Down: 941
OCCIPITAL LOBE
Volcano Plot: AD vs Control (HGNC symbols)
Heatmap Top 20 genes
E (1)
CA1
CA2
NX
1032
CA3
CA4
(3)
COX8A
C4orf48
NAA38
RPL27
NDUFA11
PMS1
HNRNPH1
TRPM7
ZNF654
LCORL
Downregulated
Upregulated
log2 Fold Change
(2)
CA1
CA2
NX
780
CA3
CA4
(4)
Condition
Control
AD
ARHGAP33
RPUSD1
POLR2J
XYLT2
TRAPPC6A
SPPL2B
ARX
C19orf60
WDR75
HMGN5

Supplementary Figure S1. Differential expression across multiple brain regions

A, B, C, and **D**. Schematic overview of the region of the samples of dataset GSE67333, GSE95587, GSE159699, and GSE203206 *Center*: Volcano plot showing differential expression between AD and control groups (upregulated genes in blue; downregulated genes in red). *Right*: Heatmap of the top 20 differentially expressed genes for each dataset. **E**. The GSE278723 dataset comprised transcriptomic profiles from hippocampal subregions (CA1–CA4) and the entorhinal cortex. (1) and (2) Venn diagram showing the overlap of up- and down- regulated genes from the five regions respectively. (3). Volcano plot showing gene differential expression (AD vs. Control) after data collapsed to one value per gene from the five regions, x-axis represents $\log_2$ fold change, and the y-axis represents $-\log_{10}(P$ value). **Red**: Upregulated genes; **Blue**: Downregulated genes. (4). Heatmap of the top 20 most differentially expressed genes from the merged common UP and DOWN gene sets. The colour scale represents relative expression (blue = low, white = intermediate, red = high).

Abbreviations: CA: cornu ammonis, NX: Entrorhinal cortex

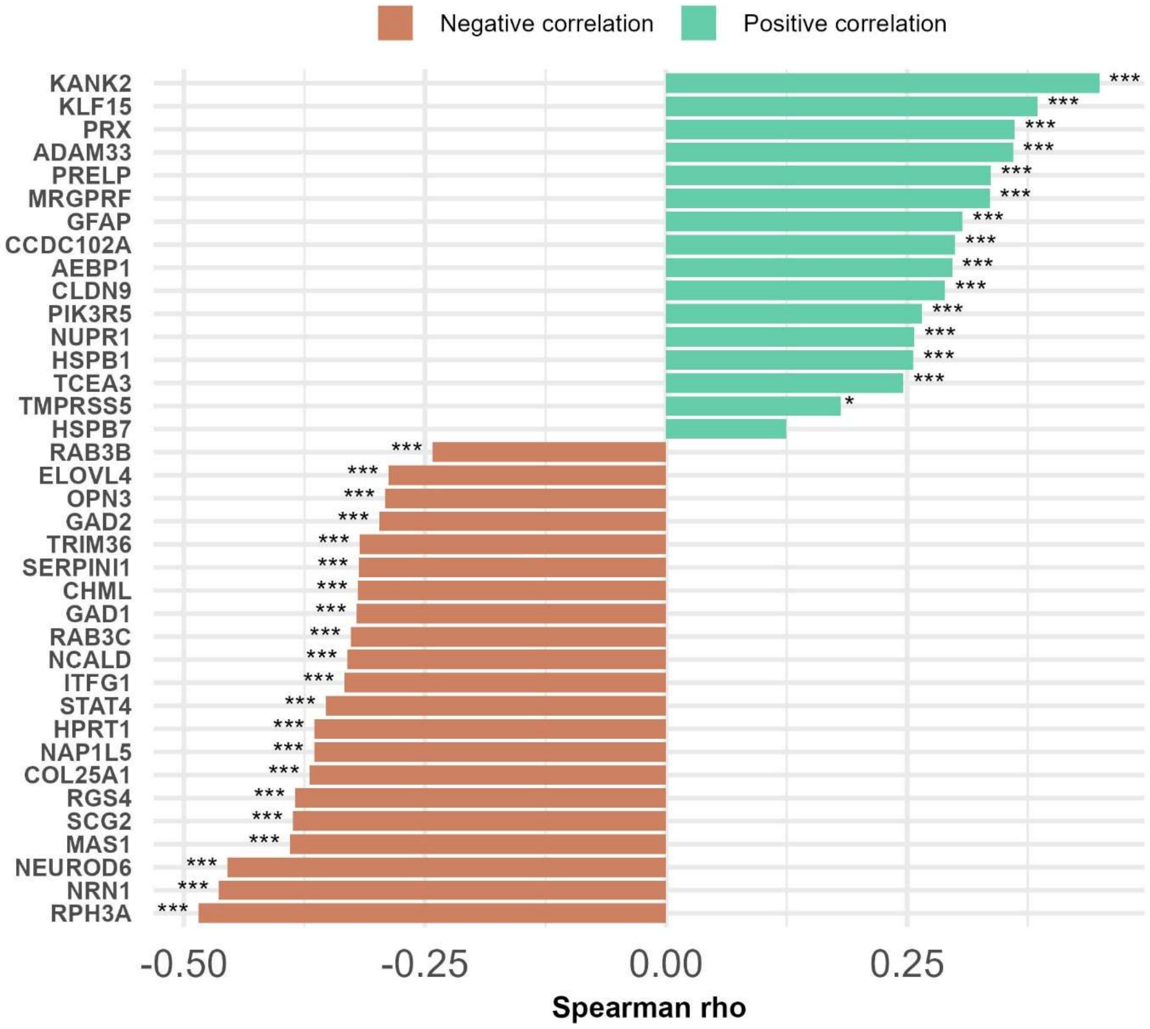

Supplementary Figure S2. Spearman correlations between consensus gene expression and Braak stage.

Bar plot showing Spearman correlation coefficients (ρ) between ComBat-corrected, z-score normalized expression of each of the 37 consensus genes and numeric Braak stage (0–6) across 201 samples. Green bars indicate positive correlations (expression increases with Braak stage); brown bars indicate negative correlations (expression decreases with Braak stage). Genes are ordered by Spearman ρ. Asterisks denote statistical significance after FDR correction (*FDR ≤ 0.05, **FDR ≤ 0.01, ***FDR ≤ 0.001).
